# The use of cervical cerclage in asymptomatic twin pregnancies with cervical shortening or dilation: a twelve-year retrospective cohort study

**DOI:** 10.1186/s12884-023-06013-6

**Published:** 2023-09-29

**Authors:** Hongying Tan

**Affiliations:** 1 Zhejiang College of Security Technology, 325027 Wenzhou, China; 2 Department of Obstetrics and Gynecology, the Second Affiliated Hospital of Wenzhou Medical University, Wenzhou, China

**Keywords:** Cervical cerclage, Twin pregnancy, PTB, Cervical length, Cervical dilation

## Abstract

**Background:**

To identify the effect and optimal time of cervical cerclage in asymptomatic twin pregnancies with cervical shortening or dilation.

**Methods:**

This observational retrospective study enrolled all women with asymptomatic twin pregnancies who were diagnosed with asymptomatic cervical shortening or dilation at the Second Affiliated Hospital of Wenzhou Medical University between 2010 and 2022. Women included were allocated into the cerclage group (n = 36) and the no cerclage group (n = 22). The cerclage group was further divided into the cerclage group (< 24 weeks group) and the cerclage group (24–28 weeks group) according to the time of cerclage. The no cerclage group was further divided into no cerclage group (< 24 weeks group) and no cerclage group (24–28 weeks group) according to the time of ultrasound-indicated or physical exam indicated cerclage. The rates of PTB < 24, 28, 32 and 34 weeks of gestation, maternal and neonatal outcomes were compared among the groups.

**Results:**

The gestational age (GA) at delivery was higher (*P* = 0.005) and the interval time between the presentation of the indicated cerclage and delivery was longer in the cerclage group (*P* < 0.001). The rates of PTB before 28, 32, and 34 weeks of gestation, caesarean section and stillbirth were lower in the cerclage group (*P* < 0.05). The birthweight of the twins was higher in the cerclage group (*P* = 0.012). Admissions to the NICU were more frequent in pregnancies with no cerclage (*P* = 0.008). Subgroup analysis showed that the interval time between the presentation and delivery was longer in the cerclage group (< 24 weeks) (*P* < 0.001). The GA at delivery and the birthweight of the twins were significantly higher in the cerclage group (< 24 weeks) (*P* < 0.001). No differences were found in the GA at presentation, the GA at delivery, the interval time between the presentation to delivery and birthweight between the cerclage group (24–28 weeks group) and the control group (24–28 weeks group) (*P* > 0.05).

**Conclusions:**

Cerclage appears to prolong the GA at delivery and the interval time between the presentation to delivery, and may reduce the incidence of PTB before 28, 32 and 34 weeks of gestation and adverse perinatal outcomes in asymptomatic twin pregnancies with cervical shortening or dilation. Cerclage before 24 weeks of gestation showed longer GA at delivery, longer interval time between the presentation to delivery and higher birthweight of the twins. The GA at presentation, the GA at delivery, the interval time between the presentation to delivery and birthweight in women with cerclage at 24–28 weeks were similar to those in women without cerclage at 24–28 weeks.

## Background

Globally, the incidence of twin pregnancies has increased over the past 20 years, mainly because of the rapid development of assisted reproductive technologies (ART) and gonadotropin stimulation protocols [1]. Over 30% of babies born with very low birth weight are twins, and they cause over 20% of infant deaths [2]. Preterm birth (PTB), which is defined as delivery between 28 and less than 37 weeks of gestation, is a significant cause of neonatal morbidity and mortality including neurodevelopmental disorders, respiratory infections and gastrointestinal diseases [3–5]. Very premature births, which is defined as neonates born before 33 weeks of gestation, has accounted for nearly a third of PTB and lead to a great cost to health services [4]. Therefore, preventing the occurrence of PTB is an important healthcare priority.

There are a number of risk factors for PTB in twin pregnancies including intrauterine infection, preeclampsia, cervical insufficiency, increased uterine distension and a history of PTB [6]. The cervical insufficiency is a well-recognized cause of mid-trimester miscarriage, recurrent pregnancy loss in the mid-trimester, and preterm labor presenting with bulging membranes in the absence of significant uterine contractility or rupture of membranes [7].The incidence of cervical insufficiency in twin pregnancies has been reported to be significantly higher than that in women with singleton pregnancies. Cervical cerclage is a surgical technique applied to strengthen the weak cervix, maintain its length and preserve the mucus plug at the cervical opening, thus protecting against ascending infection [8]. However, the effectiveness and safety of cervical cerclage in the treatment for cervical insufficiency in twin pregnancies remain controversial based on limited data [9]. Several studies have found that cerclage for women with cervical dilation or cervical shortening is beneficial for preventing PTB and improving perinatal mortality and morbidity in singleton gestations [10–12]. On the contrary, some researchers opposed cervical cerclage in twin pregnant women with owing to some complications such as increasing the risk of PTB [13]. Additionally, few studies have compared the efficacy of cervical cerclage at different gestational weeks.

Therefore, the purpose of this study was to identify the effect of cervical cerclage on preventing PTB and improving maternal and neonatal outcomes in asymptomatic twin pregnancies with cervical shortening or dilation. Additionally, we aimed to determine the optimal time of cervical cerclage by comparing the perinatal outcomes of cervical cerclage performed at different gestational age.

## Methods

This was a retrospective cohort study enrolled all the twin pregnant women with asymptomatic cervical shortening or dilation before 28 weeks gestation at the Second Affiliated Hospital of Wenzhou Medical University from January 1, 2010 to September 30, 2022. Pregnant women aged 18–50 years with complete medical records and those with asymptomatic cervical dilation, visible membranes by speculum exam or with short cervical length (CL) ≤ 25 mm by transvaginal ultrasound were eligible to participate in this study. The exclusion criteria were women with chronic hepatic, cardiac or renal disease, chronic hypertension or diabetes, monochorionic-monoamniotic pregnancy, single intrauterine fetal demise, multifetal pregnancy reduction, preterm premature rupture of membranes (PPROM) prior to randomization, twin-twin transfusion syndrome, placental abruption or placenta previa, active uterine contractions, clinical chorioamnionitis or active vaginal bleeding at the time of diagnosis, evidence of life-incompatible fetal anomalies and cerclage already in place.

All eligible women were extensively informed about the possible advantages and risks of conservative management and cervical cerclage for asymptomatic cervical shortening or dilation. The treatment plan was agreed based on the women’s preferences. Vaginal progesterone was provided to women in both the cerclage and the no cerclage groups to prevent preterm labor who received vaginal progesterone suppositories in a dose of 400 mg daily till 37 weeks of gestation. Then women included were categorized into the cerclage group and the control group (no cerclage group). The cerclage group was further divided into the cerclage group (< 24 weeks group) and the cerclage group (24–28 weeks group) according to the time of cerclage. The control group was further divided into the control group (< 24 weeks group) and the control group (24–28 weeks group) according to the time of ultrasound-indicated or physical exam indicated cerclage.

The procedure of cerclage was performed using the McDonald technique by the chief obstetricians who had the similar operative technique at our tertiary institution. All the pregnant women with cerclage received prophylactic perioperative antibiotics (cephalosporin) and tocolytics (ritodrine), as well as routine prenatal examinations until delivery. The cervical cerclage was removed at 37 weeks gestation or during an elective cesarean section or in case of PPROM, active uterine contraction, or evidence of chorioamnionitis. In the control group, bed rest and keeping the bowels open were recommended. If contraction was observed, tocolytic therapy was administrated. Data on demographic characteristics, maternal and neonatal outcomes of the included women were collected from the medical records. Demographic characteristics included maternal age, nulliparous, smoking status, use of ART, chorionic, CL ≤ 25 mm, number of previous uterine instrumentation, previous obstetric history such as abortion, PTB or prior loop electrosurgical excision procedure (LEEP). Maternal outcomes included gestational age (GA) at the time of presentation of cervical shortening or dilation, GA at delivery, interval time from presentation to delivery, spontaneous PTB (SPTB), preterm premature rupture of membranes (PPROM), intrauterine infections, mode of delivery and stillbirth. Neonatal outcomes included birthweight, Apgar at 5 min < 7, low birth weight (< 2500 gram), very low birth weight (< 1500 gram), admission to neonatal intensive care unit (NICU), respiratory distress syndrome (RDS), intraventricular hemorrhage (IVH), necrotizing enterocolitis (NEC), sepsis, retinopathy of prematurity (ROP), neonatal mortality and bronchopulmonary dysplasia. GA was determined by last menstrual period, embryo transplant time and craniocaudal length calculated by ultrasonography during the first trimester of pregnancy. All women underwent ultrasound examination by an experienced operator to determine chorionicity and fetal viability, fetal abnormalities, and CL.

The primary outcome was the incidence of SPTB < 34 weeks of gestation. Secondary outcomes included the incidence of SPTB < 32, < 28 or < 24 weeks of gestation, perinatal mortality and composite neonatal adverse outcomes which required at least one of the following: RDS, IVH, NEC or ROP.

### Statistical analyses

SPSS 22.0 software (SPSS Inc., Chicago, IL, USA) was used for statistical analysis. Data for normally distributed continuous variables were shown as mean ± standard deviation (SD), and date for non-normally distributed continuous variables were presented as the median (interquartile range). Categorical variables were written as number (percentage). Univariate comparisons of dichotomous data were performed with the chi-square test or Fisher’s exact test with continuity correction. Comparisons between the two groups for normally distributed continuous variables were performed with the T-test, and with the Wilcoxon and Mann-Whitney U tests or non-parametric test was used for non-normally distributed continuous variables. All *P*-values were two-sided, and if below 0.05 the results were considered statistically significant.

## Results

**Fig. 1:**
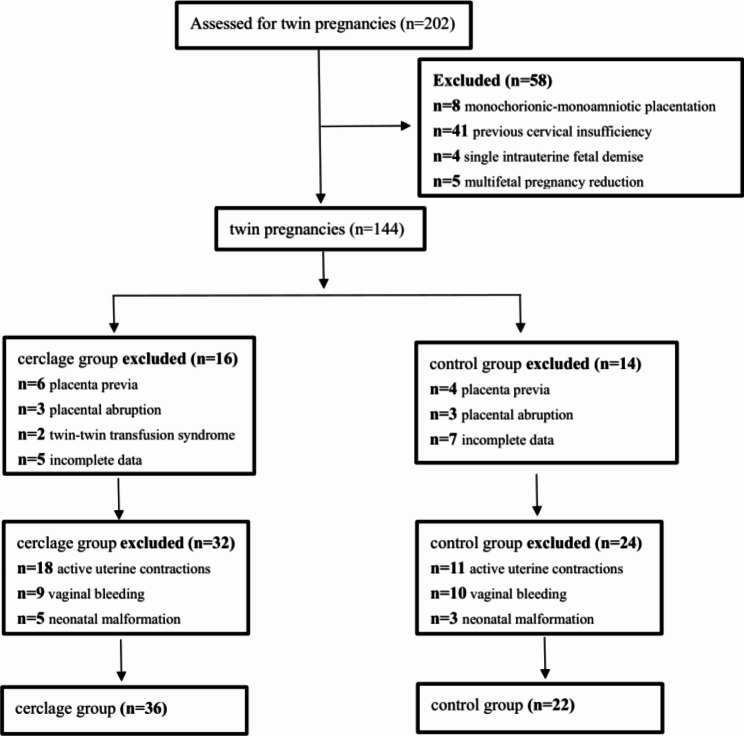
Flow diagram showing the study population of twin pregnancies included in the study

As shown in Fig. 1, a total of 202 pregnant women with diamniotic twin pregnancies were enrolled between 2010 and 2022, of whom 144 were excluded for the following reasons: monochorionic-monoamniotic placentation (n = 8), previous cervical insufficiency (n = 41), single intrauterine fetal demise (n = 4), multifetal pregnancy reduction (n = 5), placenta previa(n = 10), placental abruption (n = 6), twin-twin transfusion syndrome (n = 2), incomplete data (n = 12), active uterine contractions (n = 29), vaginal bleeding (n = 19), neonatal malformation (n = 8). Finally, 58 women with twin pregnancies remained and were divided into cerclage group (n = 36) and control group (n = 22) (Fig. 1).

There were no statistically significant differences in maternal age, nulliparous, smoking status, number of previous uterine instrumentation, IVF, IUI, chorionic, previous obstetric history including abortion, PTB or LEEP between the cerclage group and the control group (*P* > 0.05) (Table 1). No significant difference was found in GA at the time of presentation of cervical shortening or dilation (21.61 ± 3.1 vs. 22.77 ± 2.3 weeks, *P* > 0.05) (Table 2). Compared with the control group, the incidence of SPTB < 34 weeks (55.6% vs. 88.4%, *P* < 0.05), < 32 weeks (50.0% vs. 81.8%, *P* < 0.05), and < 28 weeks (22.2% vs. 54.5%, *P* < 0.05) in the cerclage group significant decreased and the interval from presentation to delivery was significantly longer in the cerclage group (9.75 ± 5.2 vs. 4.14 ± 3.1 weeks, *P*<0.05) (Table 2). GA at delivery was significantly later in the cerclage group than that in the control group (31.06 ± 4.9 vs. 27.05 ± 5.5 weeks, *P*<0.05) (Table 2). Besides, the rate of caesarean section was higher and the rate of vaginal delivery was lower in the cerclage group when compared to the control group (*P*<0.05, Table 2). However, the rate of stillbirth was higher in the control group (*P*<0.05, Table 2). There were no significant differences in the rates of PPROM and intrauterine infection between the two groups (Table 2).

**Fig. 2:**
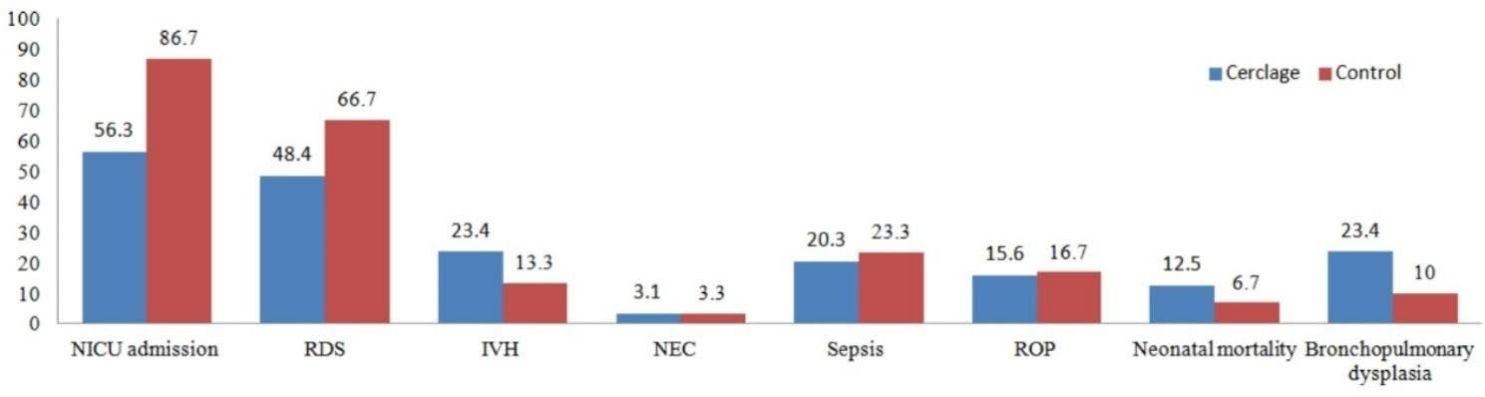
Neonatal morbidity and mortality in two groups

Neonatal outcomes of the live newborns were presented in Table 3. Neonates in the cerclage group had a higher birthweight than those in the control group (1862 ± 772 vs. 1485 ± 606 gram, *P*<0.05). The proportion of live newborns admitted to NICU was lower in the cerclage group than that in the control group (56.3% (36/64) vs. 86.7% (26/30), *P*<0.05). The incidence of RDS was 48.4% and 66.7% in the cerclage group and the control group, respectively, which showed little difference (*P* > 0.05). The rates of Apgar at 5 min < 7, low birth weight (< 2500 gram), very low birth weight (< 1500 gram), IVH, NEC, sepsis, ROP, neonatal mortality, and bronchopulmonary dysplasia were all low in both two groups, and no statistical significance were found between two groups (*P* > 0.05, Fig. 2).

The results of maternal and neonatal outcomes of the two subgroups (< 24 weeks group and 24–28 weeks group) in the cerclage group and the control group were presented in Table 4. GA at the time of presentation of cervical shortening or dilation showed little differences between the cerclage group (< 24 weeks) and the control group (< 24 weeks) (*P* > 0.05). Compared with the control group (< 24 weeks group), women in the cerclage group (< 24 weeks group) had higher GA at delivery and longer interval from presentation to delivery (*P*<0.05). The birth weight in the cerclage group (< 24 weeks group) was also higher than that in the control group (< 24 weeks group). However, there were no significant differences in GA at the time of presentation of cervical shortening or dilation, GA at delivery, interval from presentation to delivery and birth weight between the cerclage group (24–28 weeks group) and the control group (24–28 weeks group) (*P* > 0.05).

## Discussion

In our study, pregnant women with an ultrasound-indicated or physical exam indicated cerclage showed lower incidence of SPTB < 34 weeks, < 32 weeks, < 28 weeks, and delivered later than those with conservative management. The latency period from diagnosis of dilated cervix to delivery in the cerclage group was longer than that in the control group. Compared to the control group, the rate of caesarean section in the cerclage group was higher, while the rates of vaginal delivery and stillbirth were lower. Twins born in the cerclage group had higher neonatal birthweight and were less likely to be admitted to NICU. Additionally, our study found cervical cerclage applied before 24 weeks was more effective than that applied between 24 and 28 weeks, appearing as later GA at delivery, longer interval from presentation to delivery and higher birthweight.

A normal CL has strong stretching properties and provides supporting and defensive functions during pregnancy [14]. Several previous studies demonstrated that the risk of SPTB was negatively associated with CL [15–17]. The degree of cervical shortening had shown a great predict value of SPTB at < 32 weeks [18]. The practice of cervical cerclage, as an alternative treatment in twin pregnancies with short cervix, remains controversial. Some early studies reported that cerclage increased the risk of PTB [19, 20], while some recent studies found that cervical cerclage reduced PTB rates and improved neonatal outcomes in twin pregnancies [21–23]. Results of our study were consistent with the latter one, we found that cervical cerclage was associated with a 35.6% reduction in the rate of SPTB < 34 weeks’ gestation in twins with asymptomatic cervical shortening or dilation. The women in the cerclage group had lower rates of SPTB < 32, < 28 weeks and delivered later than those in the control group. This was probably because cervical cerclage might prevent the extension of the lower uterine segment and the expansion of the cervical internal os. by strengthening the tension of the cervical canal and the intracervical surgical system might improve its ability to bear the load of the fetus and its appendage during the third trimester of pregnancy [24]. Therefore, cerclage would be recommended if evidence of CL shortening to 25 mm or less is detected on ultrasound. The differences between our results and the former one could be partly explained by the different population characteristics [25].

The process from cervix shortening to cervix dilating is continuous and takes days or weeks [8]. The association between transvaginal-ultrasound indicated asymptomatic cervical shortening or dilation and physical examination has been assessed. A recent meta analysis showed that vaginal progesterone may play a role in reducing the rates of preterm birth and neonatal morbidity and mortality in twin gestations with sonographic short cervix [26]. Therefore, all the eligible women included in our study were routinely provided with vaginal progesterone to prevent preterm birth. Groom KM et al. found that the amniotic membrane was visible in 67% of patients with CL ≤ 10 mm and in 20% of patients with CL ≤ 20 mm [27]. A recent study demonstrated that asymptomatic cervical dilation in women without prior PTB but with short cervix was associated with poor perinatal outcomes [28]. Thus, physical exam should be considered in women with short cervix and prevention measures should be made to improve perinatal outcomes.

A retrospective study concentrating on women who underwent physical examination-indicated cerclage in twin pregnancies showed that the time from diagnosis of cervix shortening or dilating to delivery was significantly longer and the incidence of SPTB was significantly decreased at any given GA, along with a significant reduction in perinatal mortality and morbidity [29]. Similarly, our data showed that women with transvaginal-ultrasound or physical examination-indicated cerclage had later delivery age, longer latency period from diagnosis to delivery, higher neonatal birthweight, lower NICU admission rate and stillbirth rate than those with conservative management. This might be explained by the decreased rate of PTB in the cerclage group. However, in our study, cesarean section rates were found to be higher in the cerclage group. The fear of vaginal birth failure, combined with maternal preference for cesarean delivery and aversion to prolonged labor may influence pregnant women with cerclage group to choose cesarean delivery rather than vaginal delivery. The clinical practice guideline was updated by the Society of Obstetricians and Gynaecologists of Canada (SOGC)’s Maternal Fetal Medicine Committee in 2019, and stated that rescue cerclage in multiple gestations is of potential value and should be considered when the cervix is dilated to > 1 cm [30]. In conclusion, whether ultrasound-indicated cerclage or physical exam indicated cerclage was a beneficial treatment for women with twin pregnancies, which can prolong gestational weeks and improve neonatal survival.

In addition, the optimal gestational age for cervical cerclage in twin pregnancies is uncertain. Most of the studies focused on cerclage performed before 24 weeks [31–34]. A few studies focused on cerclage performed before 26 weeks [35, 36]. So far, there were few studies focused on cerclage performed between 24 and 28 weeks. Both these studies and ours found that cerclage before 24 weeks in twin pregnancies was associated with significantly longer gestational age, decreased incidence of SPTB and improved perinatal outcomes. However, the results of subgroup analysis in our study showed that there were no differences in GA at delivery, presentation to birth interval, and neonatal birthweight between cerclage group and control group when cervical cerclage was placed between 24 and 28 weeks. Similarly, a study led by Moti et al. elaborated on the undefined role of cerclage that was performed between 24 and 26 weeks in singleton pregnancies [37]. Therefore, cervical cerclage placed before 24 weeks is recommended in twin pregnancies, while cerclage placed between 24 and 28 weeks should be cautious.

The strengths of our study were as follows. First, the data were collected at a tertiary hospital, where all the cerclages were placed by the chief obstetricians, thus avoiding inconsistencies in technique, experience, or management differences between hospitals. Second, to our knowledge, this was the first time to perform a subgroup analysis of gestational age for the procedure to find the optimal weeks of cerclage. The limitations of this study were the relatively small sample size, the retrospective design and associated caveats. A larger sample size may allow us to better match or stratification of propensity scores, dividing subjects into different categories of cervical insufficiency, which may provide greater insight into which groups of women would benefit most from cerclage. Additionally, further clinical trials are really important. The prospective study (NCT03340688), which is performed to investigate the effectiveness of cervical cerclage in asymptomatic twin pregnancies, should be concerned. Therefore, further randomized control studies with large samples are needed to evaluate the efficacy of cerclage for women with twin pregnancies.

## Conclusions

The use of cervical cerclage appeared to reduce the rates of PTB before 28, 32 and 34 weeks of gestation and adverse perinatal outcomes in asymptomatic twin pregnancies with cervical shortening or dilation. Cerclage before 24 weeks of gestation may play an important role in prolonging the gestational age at delivery, extending the interval time from the presentation to delivery and increasing birthweight of the twins, while cerclage between 24 and 28 weeks of gestation was ineffective. Therefore, cerclage, especially those performed before 24 weeks of gestation, might be preferred in twin pregnancies with asymptomatic cervical shortening or dilation.

**Table 1:** Maternal demographics of the cerclage and control groups

Characteristic	Cerclage group (n = 36)	Control group (n = 22)	*p* value
Maternal age (weeks) (mean ± SD)	31.30 ± 4.6	31.01 ± 3.4	0.123
Nulliparous, n(%)	29 (80.6)	20 (90.9)	0.2495
Smoking n(%)	0(0)	0(0)	NA
**Number of previous uterine instrumentation** n(%)			0.315
0	23(63.9)	13(59.1)	
1	8(22.2)	8(36.4)	
≥ 2	5(13.9)	1(4.5)	
IVF n(%)	30(83.3)	16(72.7)	0.333
IUI n(%)	1(2.8)	0(0)	1.000
**Chorionic**			1.000
Dichorionic-diamniotic, n(%)	34(94.4)	20(90.9)	
Monochorionic-diamniotic n(%)	2(5.6)	2(9.1)	
CL ≤ 25 mm n(%)	24(66.7)	13(59.1)	0.560
**Previous obstetric history**			
Abortion, n (%)	26(72.2)	11(50.0)	0.088
Preterm birth	1(2.8)	1(4.5)	1.000
Prior LEEP	0(0)	0(0)	NA

**Table 2:** Delivery outcomes of twin pregnancies in the cerclage group and control group

Characteristic	Cerclage group n = 36	Control group n = 22	*p* value
GA at presentation (mean ± SD)	21.61 ± 3.1	22.77 ± 2.3	0.133
GA at delivery (mean ± SD)	31.06 ± 4.9	27.05 ± 5.5	0.005
Presentation to birth interval (mean ± SD)	9.75 ± 5.2	4.14 ± 3.1	< 0.001
SPTB < 34 weeks n(%)	20(55.6)	19(86.4)	0.033
SPTB < 32 weeks n(%)	18(50.0)	18(81.8)	0.032
SPTB < 28 weeks n(%)	8(22.2)	12(54.5)	0.012
SPTB < 24 weeks n(%)	3(8.3)	5(22.7)	0.250
PPROM n(%)	14(38.9)	10(45.5)	0.622
Intrauterine infection n(%)	12(36.1)	9(40.9)	0.560
**Mode of delivery** n(%)			
Caesarean section	22(61.1)	7(31.8)	0.016
Vaginal delivery	14(38.9)	15(68.2)	
Stillbirth n(%)	8(11.1)	14(31.8)	0.006

**Table 3:** Neonatal outcomes

Outcome (Live-born twins only)	Cerclage group n = 64	Control group n = 30	*p* value
Birthweight (gram)	1862 ± 772	1485 ± 606	0.012
Apgar at 5 min < 7	1(1.8)	0(0)	1.000
Low birth weight (< 2500 gram)	43(67.2)	24(80.0)	0.201
Very low birth weight (< 1500gram)	25(39.1)	16(53.3)	0.193
NICU admission	36(56.3)	26(86.7)	0.008
composite adverse outcomes			
RDS	31(48.4)	20(66.7)	0.098
IVH	15(23.4)	4(13.3)	0.255
NEC	2(3.1)	1(3.3)	1.000
Sepsis	13(20.3)	7(23.3)	0.739
ROP	10(15.6)	5(16.7)	0.898
Neonatal mortality	8(12.5)	2(6.7)	0.620
Bronchopulmonary dysplasia	15(23.4)	3(10.0)	0.207

**Table 4:** The subgroup of women with < 24 weeks and 24–28 weeks in two groups

	< 24 weeks			24–28 weeks		
	Cerclage group(n = 25)	Control group (n = 12)	*p* value	Cerclage group (n = 11)	Control group (n = 10)	*p* value
GA at presentation	20.20 ± 2.6	21.00 ± 1.3	0.220	24.82 ± 0.9	24.90 ± 1.0	0.843
GA at delivery	30.48 ± 5.2	23.42 ± 4.0	< 0.001	32.36 ± 4.0	31.40 ± 3.6	0.569
Presentation to birth interval	10.72 ± 5.5	3.17 ± 2.6	< 0.001	7.55 ± 3.9	5.30 ± 3.4	0.178
Birthweight	1869 ± 827	1079 ± 313	0.000	1848 ± 673	1688 ± 620	0.429

## Data Availability

The datasets generated and/or analysed during the study are available from the corresponding author on reasonable request.

## Abbreviations

CL: cervical length
GA: gestational age
IVF: in vitro fertilization
IUI: intra-uterine insemination
LEEP: loop electrosurgical excision procedure
NICU: neonatal intensive care unit
NEC: necrotizing enterocolitis
PPROM: preterm premature rupture of membranes
PTB: preterm birth
RDS: respiratory distress syndrome
ROP: retinopathy of prematurity
SD: standard deviation
SPTB: spontaneous preterm birth
IPDMA: individual patient data meta-analyses
RCTs: randomized controlled trials
